# Association between depressive symptoms and physical exercise in college students: longitudinal mediating effects of social support and psychological resilience

**DOI:** 10.3389/fpsyg.2025.1598936

**Published:** 2025-06-23

**Authors:** Yuanyuan Hou, Xinyi Liu, Zhendong Li, Shouzhen Huang, Wenjuan Chen, Yuhao Jiang, Panpan He, Jingquan Sun, Haijun Han

**Affiliations:** ^1^Institute of Sports Science, Sichuan University, Chengdu, China; ^2^Paotongshu Primary School, Sichuan Province, Chengdu, China

**Keywords:** physical exercise, depressive symptoms, social support, psychological resilience, cross-lagged study, mediating effects

## Abstract

**Purpose:**

This longitudinal study aimed to explore the bidirectional causal relationship between depressive symptoms and physical exercise among college students, with a focus on the mediating roles of social support and psychological resilience.

**Methods:**

The Self-Rating Depression Scale (SDS) was used to assess depression. Physical activity Rating Scale (PARS-3), Perceived Social Support Scale (PSSS), Connor-Davidson Resilience Scale (CD-RISC), A longitudinal study of 1,413 college students was conducted over three months, collecting data at two time points (T1: September 15, 2024; T2: 17 December 2024). In this longitudinal study, 1,350 valid samples were obtained. Primary analyses included independent samples t-test, chi-square test, and Pearson correlation. Amos 28.0 was used to construct the cross-lag model and structural equation model for analysis.

**Results:**

(1) Depressive symptoms at T1 negatively predicted physical activity at T2 (*β* = −0.07, *p* < 0.05). Physical exercise at T1 negatively predicted depressive symptoms at T2 (*β* = −0.08, *p* < 0.05), and depressive symptoms and physical exercise could predict each other. (2) Social support at T2 played a delayed mediating role in depressive symptoms at T1 and physical exercise at T2 (*β* = −0.02, [−0.049, −0.006], *p* < 0.01). (3) Resilience at T2 played a delayed mediating role in depressive symptoms at T1 and physical exercise at T2 (*β* = −0.03, [−0.049, −0.002], *p* < 0.01). (4) Social support and resilience at T2 played a delayed chain mediating role in depressive symptoms and physical exercise at T1 (*β* = −0.01, [−0.024, −0.005], *p* < 0.01).

**Conclusion:**

This study highlights the importance of social support and resilience in promoting physical exercise and alleviating depressive symptoms in college students, as well as the interrelationship between depressive symptoms and physical exercise. The results showed that depressive symptoms not only directly affected physical exercise of college students, but also indirectly affected physical exercise of college students through the independent mediating effect of social support and psychological resilience and the chain mediating effect of social support-psychological resilience. In practice, we should improve the social support system and improve the psychological resilience of college students, which can effectively alleviate the depressive symptoms of college students and enhance physical exercise.

## 1 Introduction

In recent years, mental health issues among college students have become increasingly prominent, with depressive symptoms emerging as a critical challenge affecting academic performance, social interactions, and quality of life. Multiple studies indicate that approximately 20–30% of college students experience varying degrees of mental health issues, with depressive symptoms being particularly prevalent (Wang et al., 2025). Concurrently, physical exercise participation rates in this population remain low, as academic pressures and digitalized lifestyles exacerbate sedentary behaviors and insufficient physical activity—only 40% of college students meet recommended physical activity levels (Schmitter et al., 2020). The transition from high school to university further aggravates these issues, characterized by heightened academic demands and social changes, which increase vulnerability to mental health challenges (Tao et al., 2024). Consequently, depressive symptoms and physical inactivity are widespread among college students globally. While physical exercise has been proven effective in alleviating depressive mood (Noetel et al., 2024), the bidirectional causal relationship between depressive symptoms and physical exercise, as well as their underlying mechanisms, requires further exploration.

Existing research suggests that individuals with higher depressive symptoms often lack exercise motivation due to low mood. However, social support—such as encouragement from friends, family, or peers—can enhance their willingness to engage in physical activity (Ma et al., 2023; Templeton et al., 2025). For instance, team sports or group activities foster social interaction, thereby improving perceived social support and indirectly promoting exercise behavior. Social support not only acts as a direct mediator but also indirectly facilitates exercise adherence by enhancing psychological resilience. Individuals with strong social support are more likely to access external resources when facing stress, bolstering their ability to cope with challenges and maintain long-term exercise habits (Lin et al., 2025). Longitudinal studies reveal that social support exhibits delayed effects in the relationship between depressive symptoms and physical exercise (Cao and Luo, 2024). While depressive symptoms may temporarily reduce perceived social support, its cumulative effects over time can mitigate the inhibitory impact of depression on exercise participation. Additionally, research demonstrates that students with higher psychological resilience can sustain exercise behaviors even during depressive states by setting goals and adjusting strategies (Kuang et al., 2024). Psychological resilience often interacts with social support in a chain-like mechanism, where social support strengthens psychological resources (e.g., self-confidence and optimism), further reinforcing resilience and forming an indirect pathway of “social support → psychological resilience → physical exercise” (Wu et al., 2024). For example, family support may enhance students’ psychological resilience, enabling them to proactively address exercise-related challenges. This occurs because psychological resilience directly alleviates the negative impact of depressive symptoms on physical exercise by improving self-regulation and stress tolerance. College students with higher resilience are more likely to view exercise as a proactive coping strategy rather than avoidance behavior, thereby maintaining regular exercise patterns (Gong et al., 2023). Furthermore, psychological resilience helps individuals overcome physical fatigue or psychological barriers (e.g., self-doubt), thereby enhancing exercise intensity and frequency.

In summary, the mechanisms linking depressive symptoms, physical exercise, social support, and psychological resilience are not unidirectional but complex and dynamic. Despite existing research, gaps persist in understanding the bidirectional relationship between depressive symptoms and physical exercise, as well as the mediating roles of social support and psychological resilience. Most studies employ cross-sectional designs, limiting causal inferences. To address these limitations, this study utilizes a three-month cross-lagged model to investigate the longitudinal and bidirectional relationships between depressive symptoms and physical exercise, while testing the mediating effects of social support and psychological resilience. The aims are to clarify predictive relationships between depressive symptoms and physical exercise among college students and to elucidate the dynamic interplay of social support and psychological resilience within these relationships. Based on theoretical and empirical foundations, the following hypotheses are proposed:

*H1*: T1 depressive symptoms negatively predict T2 physical exercise.

*H2*: T1 physical exercise negatively predicts T2 depressive symptoms.

*H3*: Depressive symptoms and physical exercise mutually predict each other.

*H4*: T2 social support mediates the longitudinal relationship between T1 depressive symptoms and T2 physical exercise.

*H5*: T2 psychological resilience mediates the longitudinal relationship between T1 depressive symptoms and T2 physical exercise.

*H6*: T2 social support and T2 psychological resilience exhibit a delayed chain-mediating effect between T1 depressive symptoms and T2 physical exercise.

## 2 Participants and methods

### 2.1 Participants

This study targeted undergraduate students from Sichuan University. A cluster sampling method was employed to recruit 1,413 participants for a questionnaire survey. The questionnaire comprised five sections: demographic information (e.g., gender, age), the Self-Rating Depression Scale (SDS), the Physical Activity Rating Scale, the Perceived Social Support Scale, and the Psychological Resilience Scale. Data collection was conducted in two waves at the university gymnasium, with a three-month interval between assessments (T1: September 15, 2024; T2: December 17, 2024). Identical questionnaires were administered to the same cohort at both time points. After data collection, 1,413 questionnaires were obtained. To ensure data authenticity and reliability, inclusion criteria were applied: (1) Questionnaires completed within 5–15 min. (2) Participants who completed both waves of assessments with matching student IDs and names. Following data filtering and matching, 1,350 valid samples were retained (attrition: 63; validity rate: 95.54%). Among them, 21 people were lost to follow-up due to dropping out of school, withdrawing from school or changing majors. 42 unqualified questionnaires were excluded (incomplete questionnaires). The final sample included: Gender: male (*n* = 902, 66.8%), female (*n* = 448, 33.2%). Academic year: freshmen (*n* = 817, 60.5%), sophomores (*n* = 525, 38.9%), juniors (*n* = 6, 0.4%), seniors (*n* = 2, 0.1%). Only-child status: yes (*n* = 609, 45.1%), no (*n* = 741, 54.9%). Residential background: urban (*n* = 941, 69.7%), rural (*n* = 409, 30.3%). The mean age of participants was 18.51 ± 0.79 years. This study adhered to the principles of the Declaration of Helsinki and was approved by the Medical Ethics Committee of Sichuan University (approval no.: K2022016). All participants provided written informed consent prior to participation.

### 2.2 Methods

#### 2.2.1 Self-rating depression scale (SDS)

Depressive symptoms were assessed using the Self-Rating Depression Scale (SDS) developed by Zung at Duke University School of Medicine in 1965 (Zung, 1965). The SDS comprises 20 items, with half positively worded and half reverse-scored. The scale includes four subdimensions: psycho-affective symptoms (2 items), somatic disturbances (8 items), psychomotor disturbances (2 items), and depressive psychological disturbances (8 items). Participants rated their emotional experiences over the past week on a 4-point Likert scale, ranging from 1 (“a little or none of the time”) to 4 (“most or all of the time”). Prior to analysis, reverse-scored items were recoded, and the total raw score was summed and converted to a standardized score by multiplying by 1.25, yielding a range of 25–100. Higher scores indicate more severe depressive symptoms. Based on prior research in Chinese populations (Liu et al., 1995; Wang et al., 2024), severity thresholds were defined as follows: SDS score <50 (no depression), 50–59 (mild depression), 60–69 (moderate depression), and 70–100 (severe depression). The SDS has demonstrated robust cross-cultural validity and reliability. In this study, Cronbach’s *α* coefficients were 0.847 (T1) and 0.854 (T2), with a test–retest reliability of 0.531, confirming adequate internal consistency and temporal stability.

#### 2.2.2 Physical activity rating scale (PARS-3)

Professor Liang Deqing’s physical activity rating Scale, which was compiled by Japanese psychologist Hashimoto Gongxio and revised by Liang et al., Wuhan Institute of Physical Education, was adopted (Liang, 1994). It mainly reflected the physical exercise of the subjects in the past month. Relevant studies in China have shown that the Chinese version of PARS can better evaluate the physical activity of ordinary college students (Cao and Luo, 2024). The Cronbach’s *α* coefficients of the two tests in this study were 0.646 and 0.746, respectively, and the test–retest reliability was 0.746. The scale involved three indicators of physical exercise intensity, time and frequency. Each indicator was scored by 5-point Likert. The intensity and frequency were scored as “1–5 points,” and the time was scored as “0–4 points.” The amount of exercise was calculated using the formula: “intensity × time × frequency,” so the scale scores range from “0 to 100. According to previous studies in China, the physical activity levels of college students were classified as follows: “≤19 as small exercise (insufficient physical activity), 20–42 as medium exercise, ≥43 as large exercise.”

#### 2.2.3 Perceived social support scale (PSSS)

The English version of the Perceived Social Support Scale was developed by Blumenthal and others in 1987 and translated into Chinese by Huang and Ren (1996). It is a social support scale that measures the individual’s self-understanding and self-feeling of social support and is widely used in Chinese college students (Lin et al., 2025). The scale included 12 items and consisted of three dimensions: family support, friend support and other support. A 7-point Likert scale was used, ranging from 1 = strongly agree to 7 = strongly agree. The total score ranged from 12 to 84, with higher scores indicating higher overall social support. In this study, the Cronbach’s *α* coefficient of the first measurement of the scale was 0.945. Family support included 4 items, and its *α* coefficient was 0.897, and the test–retest reliability was 0.848. Friend support contained 4 items, the *α* coefficient was 0.913, and the test–retest reliability was 0.845. The other support contained 4 items, and its α coefficient was 0.890, and the test–retest reliability was 0.647. The Cronbach’s *α* coefficients of social support and each dimension in the second measurement were 0.946, 0.885, 0.898 and 0.916, respectively. The Cronbach’s α coefficient of each subscale ranged from 0.890 to 0.913, indicating that it had good reliability and validity.

#### 2.2.4 Connor-Davidson resilience scale (CD-RISC)

The Resilience Scale was developed by Connor and Davidson (2003), and the Chinese version was used in this study (Yu and Zhang, 2007), which has been widely used to assess resilience among Chinese college students and adults (Yu et al., 2023). The 25-item scale is divided into three dimensions: resilience (e.g., I am not easily defeated), strength (e.g., I am proud of my achievements), and optimism (e.g., I try to look at the humorous side of things when faced with problems). A 5-point Likert scale was used, that is, from 1(not at all) to 5(absolutely), with 1 = never, 2 = rarely, 3 = sometimes, 4 = often, and 5 = all the time. The total score of the scale is 100, with higher total scores in each dimension and overall scale indicating higher resilience in each dimension and overall. The Cronbach’s *α* coefficient of the first measurement of the scale in this study was 0.938. Among them, hardiness contained 13 items, the *α* coefficient was 0.898, and the test–retest reliability was 0.662. Strength contained 8 items, the *α* coefficient was 0.856, and the test–retest reliability was 0.5. Optimism contained 4 items, and its α coefficient was 0.600, and the test–retest reliability was 0.716. In the second measurement, the Cronbach’s *α* coefficients of the scale and each dimension were 0.936, 0.894, 0.847 and 0.600, respectively. The Cronbach’s α coefficient of each subscale ranged from 0.600 to 0.898. Numerous studies have shown that the scale has good reliability and validity.

### 2.3 Statistical processing

This study used SPSS 22.0 and AMOS 28.0 for data analysis. The former was used for descriptive analysis, preliminary analysis and correlation analysis, and the latter was used to construct the cross-lag model and the mediation analysis of structural equation modeling procedures. Firstly, SPSS 27.0 was used for preliminary analysis, and common method bias test, descriptive statistics, Pearson correlation analysis and the difference analysis of each variable in time were performed on the data to comprehensively understand the relationship between sample characteristics and variables. Specifically, descriptive statistics, including means, standard deviations, and frequencies, were calculated and analyzed. Pearson correlation coefficients were calculated to examine bivariate associations between study variables. Cronbach *α* coefficients were also calculated to assess the reliability of the scale, and values greater than 0.7 were considered available for further analysis. For ease of interpretation, Pearson’s correlation coefficients were divided into five levels according to Cohen’s (2013) criterian: negligible (≤0.19), low (0.20–0.39), medium (0.40–0.59), medium-high (0.60–0.79), and high (≥0.80).

Finally, to test the hypothesized causal relationship and the mediating effect between variables, Amos 28.0 was used to construct the Cross-lagged model and structural equation model. The χ2 statistical index and root mean square error (RMSEA) were used as the absolute fitting index. The comparative fit index (CFI), Tucker-Lewis index (TLI), and goodness-of-fit index (GFI) were used as incremental fit indices. The standardized root mean square residual (SRMR) of χ2/df < 5, RMSEA < 0.08, CFI, TLI and GFI values > 0.9 was less than 0.08, indicating that the model fit was good. The bias-corrected Bootstrap method was used for testing, with a random sampling number of 1,000 (Hayes, 2017). Data are presented as mean ± standard deviation (M ± SD). The level of significance for statistical analysis was set as *p* < 0.05 for statistical difference, *p* < 0.01 for statistically significant difference, and *p* < 0.001 for statistically very significant difference.

## 3 Results

### 3.1 Test of common method biases

To test the degree to which the two sets of measurement data were influenced by common method bias, Harman’s single—factor test was used (Livingstone et al., 1997). The results showed that the variance explained by the first factor was 32.10 and 31.92% respectively, both below the critical value of 40%. This indicates that there is no serious common method bias.

### 3.2 Descriptive statistics and correlation analysis

Means, standard deviations, and correlation coefficients for depressive symptoms, social support, psychological resilience, and physical exercise are in Table 1. The two—measurement results show that T1 depressive symptoms are significantly correlated with T1 social support, T1 psychological resilience, T1 depressive symptoms, T2 depressive symptoms, T2 social support, T2 psychological resilience, and T2 physical exercise (*p* < 0.01). Depressive symptoms are negatively correlated with physical exercise, with correlation coefficients of r = − 0.131 (*p* < 0.01) at T1 and r = − 0.139 (*p* < 0.01) at T2. T1 depressive symptoms are significantly negatively correlated with T2 physical exercise behavior (r = − 0.139, *p* < 0.01), and T2 depressive symptoms are significantly negatively correlated with T1 physical exercise (r = − 0.131, *p* < 0.01). The significant correlation between depressive symptoms and physical exercise meets the prerequisite conditions for cross—lagged analysis.

**Table 1:** Descriptive statistics and correlation analysis of depressive symptoms, physical exercise, social support, and psychological resilience in two measurements (*N* = 1,350).

Variable	M ± SD	SDS (T1)	RE (T1)	SS (T1)	PE (T1)	SDS (T2)	RE (T2)	PSS (T2)	PE (T2)
SDS (T1)	33.93 ± 7.446	1							
RE (T1)	92.77 ± 14.36	−0.696**	1						
SS (T1)	68.23 ± 11.56	−0.562**	0.648**	1					
PE (T1)	53.99 ± 30.64	−0.131**	0.179**	0.089**	1				
SDS (T2)	34.25 ± 7.47	0.897**	−0.696**	−0.562**	−0.131**	1			
RE (T2)	92.20 ± 14.59	−0.696**	0.894**	0.648**	0.179**	−0.696**	1		
SS (T2)	67.68 ± 11.71	−0.556**	0.643**	0.997**	0.087**	−0.556**	0.643**	1	
PE (T2)	30.50 ± 25.15	−0.139**	0.208**	0.111**	0.915**	−0.139**	0.208**	0.108**	1

SDS, Symptoms of depression; RE, Resilience of mind; SS, Social Support; PE, Physical exercise. *p < 0. 05, **p < 0. 01, ***p < 0. 001.

### 3.3 Analysis of the basic status of depression level of college students

#### 3.3.1 Analysis of depression level of college students

Among the college students surveyed in this study, 1,087 (80.5%) had no depression (normal), 193 (14.3%) had mild depression, 65 (4.8%) had moderate depression, and 5 (0.4%) had severe depression. See Table 2 for the results.

**Table 2:** Analysis of depression levels among college students.

Depression level	Frequency	Percentage (%)
No depression	1,087	80.5
Mild depression	193	14.3
Moderate depression	65	4.8
Severe depression	5	0.4
Total	1,350	100

#### 3.3.2 Analysis of differences in depression scores among different grades of college students

As shown in Table 3, the depressive symptom scores of college students from different grades are as follows: Freshmen have a mean of 41.43 (SD = 9.25), sophomores a mean of 44.59 (SD = 9.02), juniors a mean of 50.21 (SD = 16.48), and seniors a mean of 51.88 (SD = 2.65). A one-way ANOVA reveals a significant difference in the mean depressive scores among the different grades (*F* = 16.06, *p* < 0.05).

**Table 3:** Analysis of variance in depressive scores among college students of different grades.

Grade (M ± SD)						
Grade	Freshmen (*n* = 817)	Sophomores (*n* = 525)	Juniors (*n* = 6)	Seniors (*n* = 2)	*F*	*P*
Depressive score	41.43 ± 9.25	44.59 ± 9.02	50.21 ± 16.48	51.88 ± 2.65	16.06	0.009

### 3.4 Analysis of physical exercise status of college students

#### 3.4.1 Analysis of physical exercise volume of college students

Of the college students surveyed, 360 (26.7%) engaged in high-intensity exercise, 381 (28.2%) in moderate-intensity exercise, and 609 (45.1%) in low-intensity exercise. See Table 4 for details.

**Table 4:** Physical exercise level statistics among college students.

Exercise level	Frequency	Percentage (%)
Low	609	45.1
Moderate	381	28.2
High	360	26.7
Total	1,350	100

#### 3.4.2 Analysis of differences in physical exercise volume among different grades of college students

As shown in Table 5, among the surveyed college students, freshmen, sophomores, and seniors had mean exercise scores of 28.6, 33.48, and 19.45, respectively, indicating moderate—intensity exercise. Juniors had a mean score of 51.33, indicating vigorous—intensity exercise. A one—way ANOVA showed no significant difference in exercise levels across grades (*p* > 0.05).

**Table 5:** Comparative analysis of exercise volume of college students in different grades.

Grade (M ± SD)						
Grade	Freshmen (*n* = 817)	Sophomores (*n* = 525)	Juniors (*n* = 6)	Seniors (*n* = 2)	*F*	*P*
Exercise score	28.6 ± 24.09	33.48 ± 26.34	51.33 ± 33.67	19.50 ± 6.36	5.18	0.06

### 3.5 Analysis of differences in time for each variable

Paired—samples t—tests were run on the data of depressive symptoms, social support, psychological resilience, and physical exercise from the first and second measurements. Results are in Table 6 Between the two measurements, except for stable social support scores, depressive symptom scores dropped significantly (t = −2.074, *p* = 0.038), psychological resilience scores decreased significantly (t = 1.994, *p* = 0.046), and physical exercise scores fell markedly (t = 23.538, *p* < 0.001).

**Table 6:** Analysis of differences in depressive symptoms, social support, psychological resilience and physical exercise (*N* = 1,350).

Variable	T1	T2	*t*	*p*
SDS	33.93 ± 7.446	34.25 ± 7.47	−2.074	0.038
RE	92.77 ± 14.36	92.20 ± 14.59	1.994	0.046
SS	68.23 ± 11.56	67.68 ± 11.71	1.355	0.176
PE	53.99 ± 30.64	30.50 ± 25.15	23.538	<0.001

SDS, Symptoms of depression; RE, Resilience of mind; SS, Social Support; PE, Physical exercise.

### 3.6 The cross-lagged model

**Figure 1:**
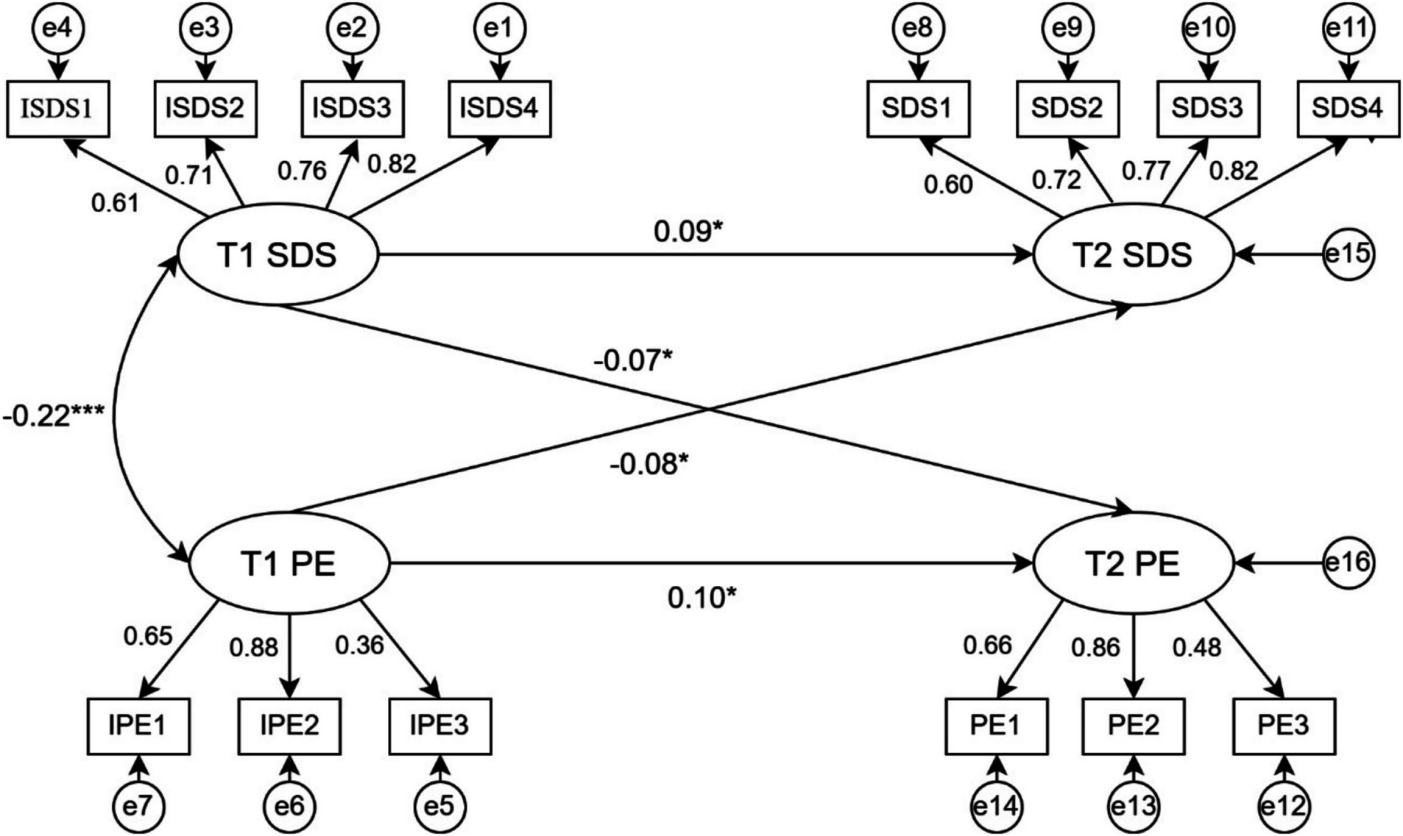
Cross-lagged model plots of depressive symptoms and physical exercise. SDS1, Psycho-affective symptoms; SDS2, Somatic disorders; SDS3, Psychomotor disorders; SDS4, Depressive disorders; PE1, Intensity of exercise; PE2, Time to exercise; PE3, Frequency of exercise. ^*^p < 0.05, ^**^p < 0.01, ^***^p < 0.001.

Based on the significant correlation between depressive symptoms and physical exercise, this study used a cross—lagged model to analyze data from two assessments three months apart, testing for bidirectional effects between depressive symptoms and physical exercise. The model was built using four T1 depressive symptom dimensions (psychotic, somatic, psychomotor, and psychological) and three T2 physical exercise dimensions (intensity, time, frequency) as indicator variables. Amos 28.0 was used with maximum likelihood estimation for model fitting. The model showed good fit indices (χ2/df = 2.42, CFI = 0.981, GFI = 0.982, TLI = 0.976, RMSEA = 0.032, SRMR = 0.04). The cross—lagged path diagram (Figure 1) shows that T1 depressive symptoms negatively predict T2 physical exercise (*β* = −0.07, [−0.140, −0.001], *p* = 0.045), supporting Hypothesis 1. Similarly, T1 physical exercise negatively predicts T2 depressive symptoms (β = −0.08, [−0.151, −0.008], *p* = 0.027), supporting Hypothesis 2. Thus, depressive symptoms and physical exercise can predict each other, supporting Hypothesis 3. The results indicate mutual negative effects between depressive symptoms and physical exercise.

### 3.7 Mediation analysis

This study used a structural equation model to test the delayed chain—mediated effects of social support and psychological resilience on the longitudinal link between college students’ depressive symptoms and physical exercise.

**Figure 2:**
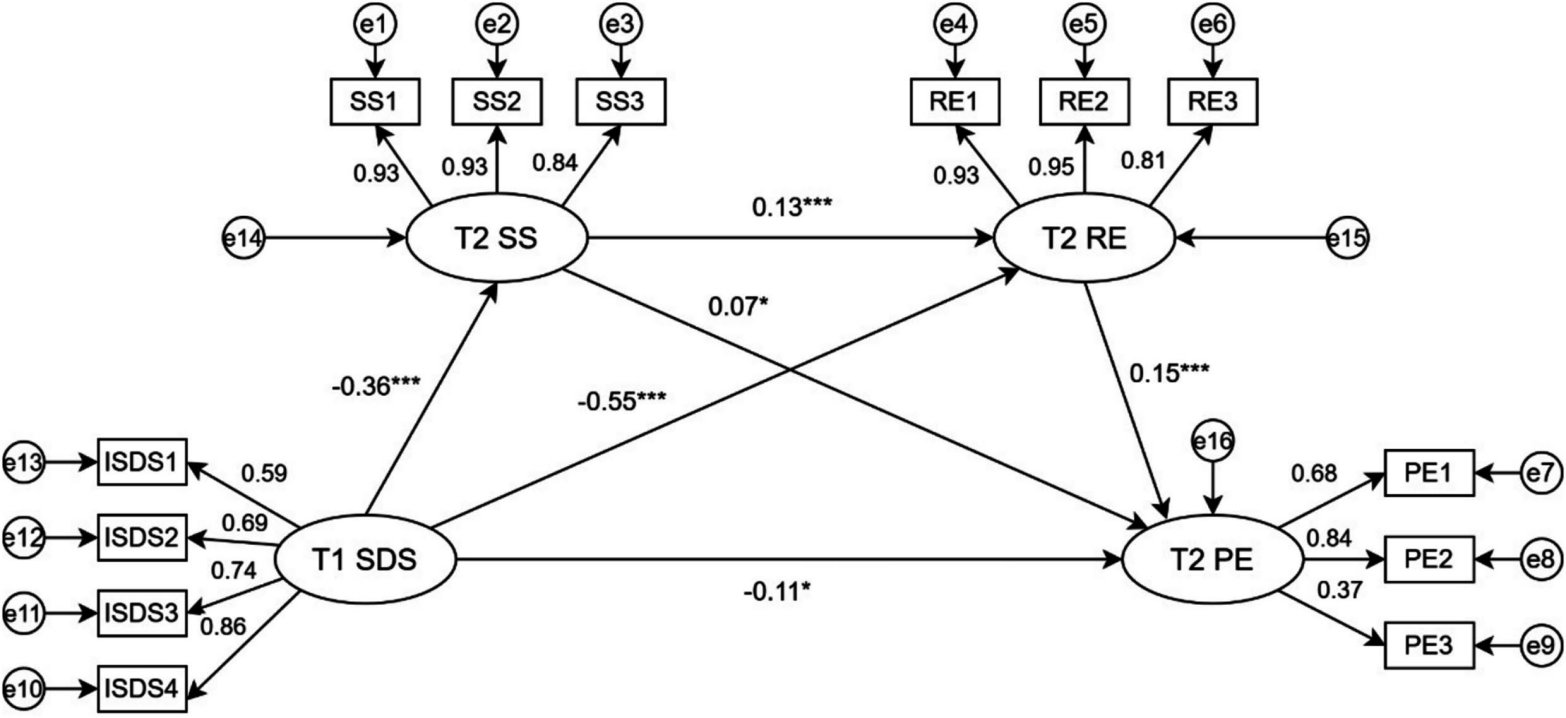
Plot of standardized path coefficients for the time-lapse chain mediation model. SDS, Symptoms of depression; RE, Resilience of mind; SS, Social Support; PE, Physical exercise; SDS1, Psycho-affective symptoms; SDS2, Somatic disorders; SDS3, Psychomotor disorders; SDS4, Depressive disorders; SS1, Family support, SS2, Support from friends; SS3, Other Support; RE1, Strength of force; RE2, Hardiness; RE3, Optimism; PE1, Intensity of exercise; PE2, Time to exercise; PE3, Frequency of exercise. *p < 0. 05, **p < 0. 01, ***p < 0. 001.

T1 depressive symptoms (four dimensions: psychotic—affective, somatic, psychomotor, and psychological) were the predictor, and T2 physical exercise (three dimensions: intensity, time, frequency) was the outcome. T2 social support (three dimensions: family, friend, and other support) and T2 psychological resilience (three dimensions: toughness, strength, and optimism) were mediators. Maximum likelihood estimation was used for model fitting. The model fit was good: χ2/df = 4.6, RMSEA = 0.05, CFI = 0.979, GFI = 0.969, TLI = 0.972, and SRMR = 0.03. The significance of the mediated effects was tested using a bias—corrected Bootstrap method with 1,000 repeated samples. Figure 2 shows the standardized path coefficients of the model.

As shown in Table 7, T1 depressive symptoms directly and negatively predict T2 physical exercise (*β* = −0.11, [−0.204, −0.010], *p* = 0.015), accounting for 47.83% of the total effect. The mediating effect of T2 social support between T1 depressive symptoms and T2 physical exercise is significant (*β* = −0.03, [−0.101, −0.002], *p* = 0.046), accounting for 13.04% of the total effect, which supports Hypothesis H4. The mediating effect of T2 psychological resilience between T1 depressive symptoms and T2 physical exercise is also significant (β = −0.08, [−0.271, −0.048], *p* = 0.008), making up 34.78% of the total effect, which supports Hypothesis H5. Moreover, the chain mediating effect of T2 social support and T2 psychological resilience between T1 depressive symptoms and T2 physical exercise is significant (β = −0.01, [−0.024, −0.005], *p* = 0.004), representing 4.35% of the total effect, thus supporting Hypothesis H6.

**Table 7:** Bootstrap results for standardized path coefficients of the model (*N* = 1,350).

Effect	Path	95%CI	β	Effect size
Direct effect	T1 SDS → T2 PE	[−0.204, −0.010]	−0.11	47.83%
Effect of mediation	T1 SDS → T2 SS → T2 PE	[−0.101, −0.002]	−0.03	13.04%
	T1 SDS → T2 RE → T2 PE	[−0.271, −0.048]	−0.08	34.78%
	T1 SDS → T2 SS → T2 RE → T2 PE	[−0.024, −0.005]	−0.01	4.35%
Indirect effect		[−0.358, −0.085]	−0.12	52.17%
Total effect		[−0.574, −0.283]	−0.23	

SDS, Symptoms of depression; RE, Resilience of mind; SS, Social Support; PE, Physical exercise.

## 4 Discussion

### 4.1 Results discussion

This study used cross-lagged and structural equation models to analyze the causal relationship between college students’ depressive symptoms and physical exercise, as well as the mediating effects of social support and psychological resilience. Over three months, from Time 1 (T1) to Time 2 (T2), both depressive symptoms and exercise levels decreased. Depressive symptoms (SDS) and physical exercise (PE) were negatively correlated, with T1 depressive symptoms negatively predicting T2 physical exercise (*β* = −0.07), and T1 physical exercise negatively predicting T2 depressive symptoms (β = −0.08). This suggests a bidirectional causal relationship where each can predict the other, indicating a dynamic interaction (Kandola and Stubbs, 2019).

This implies that depressive symptoms may reduce physical exercise participation by lowering motivation and energy, while insufficient exercise may worsen low mood, creating a vicious cycle. The neurobiological mechanisms of depression, such as reduced dopamine secretion, decrease motivation for exercise, and insufficient exercise increases inflammatory factors, further worsening mood (Kandola et al., 2019). Physical exercise enhances psychological resilience by promoting brain—derived neurotrophic factor (BDNF) secretion, which in turn promotes exercise participation (Rebar et al., 2015).

Based on Cohen and Wills (1985) buffer hypothesis, social support can reduce the negative impact of depressive symptoms on behavior like exercise by providing emotional support, resources, and a sense of belonging. This study found that T1 depressive symptoms indirectly suppress T2 physical exercise by reducing T2 social support (*β* = −0.03), confirming the Social Support Buffering Theory. Depressive symptoms may reduce perceived support by causing social withdrawal, and low social support further decreases exercise participation (Pearce et al., 2022; Yuan et al., 2025). Psychological resilience helps individuals maintain behavioral stability under stress (Hobfoll, 1989). This study shows that T1 depressive symptoms indirectly lower T2 physical exercise by depleting psychological resilience (*β* = −0.08), indicating depression may impair exercise adherence through a “resource—loss cycle.” Those with high resilience are more likely to use alternative resources to maintain exercise habits (Xu et al., 2021; Jiang and Xiao, 2024), aligning with the COR theory’s “resource—gain spiral.” Additionally, the significant chain mediating effect of social support and psychological resilience (β = −0.01) suggests depressive symptoms can indirectly inhibit exercise by reducing social support and resilience. The combined effect of psychological resilience and social support reflects the interaction between individual traits and microsystems like family and peers (Wu et al., 2025; LIU et al., 2025). Family support can enhance psychological resilience by boosting self‐efficacy, promoting exercise behavior. Social support can also strengthen resilience through resource transfer; peer encouragement helps overcome exercise challenges, accumulating resilience resources like persistence (Shah et al., 2024; Visted et al., 2024; Visted et al., 2023).

The direct effect accounts for 47.83% of the total effect, indicating other potential mechanisms, like neurobiological factors, need further exploration. Our findings align with recent longitudinal studies supporting the bidirectional relationship between depression and exercise but further reveal the chain mediating mechanism of social support and psychological resilience, addressing the limitation of previous studies focusing on single mediators. Moreover, the mediating effect of social support (13.04%) is lower than that of psychological resilience (34.78%), suggesting internal resources like resilience may be more crucial than external support for exercise behavior in college students. Other psychological factors, such as personality traits, or situational factors, such as academic stressors and environmental support, may also mediate or moderate the effects observed in our study. Future research could examine how personality traits influence an individual’s response to depressive symptoms and their subsequent engagement in physical activity. In addition, studying academic how stress either exacerbates or alleviates the relationship between depression and exercise could yield valuable insights. Environmental support, including sports facilities and university health programs, is also a potential factor that may influence this relationship. Future studies should incorporate these variables to gain a more comprehensive understanding of the complex interplay between mental health and physical activity.

### 4.2 Research significance

This research explores the bidirectional relationship between depressive symptoms and physical exercise, as well as the mediating role of social support and psychological resilience. It offers valuable insights for developing targeted interventions to enhance college students’ mental health and promote physical activity. By strengthening social support systems and building psychological resilience, universities and policymakers can effectively reduce depressive symptoms and encourage regular exercise, improving students’ overall well-being. In practice, the findings guide college mental health interventions: Universities and mental health practitioners can develop integrated programs combining social support enhancement and psychological resilience training. For example, organizing group exercise activities can promote social connections and support networks, potentially alleviating depressive symptoms and encouraging regular physical exercise. Additionally, resilience—building initiatives like stress—management workshops and cognitive—behavioral training can help students deal more effectively with academic and personal challenges. These programs can be implemented through campus recreation centers, student affairs departments, and mental health services. Furthermore, mental health practitioners can use our findings to develop targeted interventions for students with elevated depressive symptoms, focusing on enhancing psychological resilience and promoting physical activity as a supplementary treatment approach. By integrating these strategies into existing mental health programs, universities can create a more supportive environment that promotes overall student well—being and academic success.

### 4.3 Limitations and future directions

This study has some limitations despite its rigorous design. First, the sample was only from Chinese college students, which may limit the generalizability of the findings. Caution is needed when applying these results to other cultural or age groups. More diverse samples from different cultural and institutional backgrounds will be included in the future. Second, the longitudinal interval of the study was three months, a time frame chosen to take into account practical feasibility and the need to observe initial behavioral and emotional changes, however, we are aware that this may not fully capture the long-term effects of changes in psychological structure, such as the development of psychological resilience. The reliance on self-reported measures may also introduce common method bias, and future studies could use objective exercise data to enhance validity. Third, the study did not explore other potential mediators or moderators, like personality traits or environmental factors, which might influence the relationship between depressive symptoms and physical exercise.

Future research should address these limitations by incorporating diverse samples and objective measurement tools. Extending the follow-up period to over one year would help explore the long-term stability of mediating effects, and longitudinal studies with multiple time points could deepen our understanding of the dynamic relationships between these variables. Additionally, integrating multimodal data (such as physiological indicators and behavioral records) to build more comprehensive theoretical models and designing intervention experiments would help test the effectiveness of interventions targeting social support and psychological resilience.

## 5 Conclusion

This longitudinal study explores the bidirectional relationship between college students’ depressive symptoms and physical exercise, revealing a negative predictive relationship over time. Social support and psychological resilience significantly mediate this relationship, both independently and through a chain mediation model.

Theoretical contributions of this study include a deeper understanding of the interplay between mental health and physical activity, and the importance of internal and external resources. For practice, the findings highlight the need to strengthen social support systems and build psychological resilience to effectively reduce depressive symptoms and promote physical exercise among college students.

## Data Availability

The original contributions presented in the study are included in the article/supplementary material, further inquiries can be directed to the corresponding author.
